# Coating Dependent In Vitro Biocompatibility of New Fe-Si Nanoparticles

**DOI:** 10.3390/nano8070495

**Published:** 2018-07-05

**Authors:** Mihaela Balas, Florian Dumitrache, Madalina Andreea Badea, Claudiu Fleaca, Anca Badoi, Eugenia Tanasa, Anca Dinischiotu

**Affiliations:** 1 Department of Biochemistry and Molecular Biology, University of Bucharest, 91–95 Splaiul Independenţei, 050095 Bucharest, sector 5, Romania; badea_andreea08@yahoo.com (M.A.B.); ancadinischiotu@yahoo.com (A.D.); 2 National Institute for Lasers, Plasma and Radiation Physics (NILPRP), Atomistilor 409, 077125 Magurele, Romania; dumitracheflorian@yahoo.com (F.D.); claudiufleaca@yahoo.com (C.F.); anca.badoi@inflpr.ro (A.B.); 3 Department of Oxide Materials and Nanomaterials, Faculty of Applied Chemistry and Materials Science, University Politehnica of Bucharest, Gh. Polizu 1-7, 11061 Bucharest, sector 1, Romania; eugenia.vasile27@gmail.com

**Keywords:** hybrid Fe-Si nanoparticles, laser pyrolysis, Caco2 cells, cytotoxicity, oxidative stress

## Abstract

Magnetic nanoparticles offer multiple utilization possibilities in biomedicine. In this context, the interaction with cellular structures and their biological effects need to be understood and controlled for clinical safety. New magnetic nanoparticles containing metallic/carbidic iron and elemental silicon phases were synthesized by laser pyrolysis using Fe(CO)_5_ vapors and SiH_4_ gas as Fe and Si precursors, then passivated and coated with biocompatible agents, such as l-3,4-dihydroxyphenylalanine (l-DOPA) and sodium carboxymethyl cellulose (CMC-Na). The resulting magnetic nanoparticles were characterized by XRD, EDS, and TEM techniques. To evaluate their biocompatibility, doses ranging from 0–200 µg/mL hybrid Fe-Si nanoparticles were exposed to Caco2 cells for 24 and 72 h. Doses below 50 μg/mL of both l-DOPA and CMC-Na-coated Fe-Si nanoparticles induced no significant changes of cellular viability or membrane integrity. The cellular internalization of nanoparticles was dependent on their dispersion in culture medium and caused some changes of F-actin filaments organization after 72 h. However, reactive oxygen species were generated after exposure to 25 and 50 μg/mL of both Fe-Si nanoparticles types, inducing the increase of intracellular glutathione level and activation of transcription factor Nrf2. At nanoparticles doses below 50 μg/mL, Caco2 cells were able to counteract the oxidative stress by activating the cellular protection mechanisms. We concluded that in vitro biological responses to coated hybrid Fe-Si nanoparticles depended on particle synthesis conditions, surface coating, doses and incubation time.

## 1. Introduction

The biomedical applications of nanomaterials are particularly focused on magnetic nanoparticles (MNPs), due to their attractive properties, such as superparamagnetism and significant saturation magnetization. Thus, they have been successfully tested as contrast agents in magnetic resonance imaging (MRI) [1], drug or bioactive molecule carriers for direct delivery to the disease site [2,3], and hyperthermia [4]. These nanosystems were also used in a wide variety of areas, magnetic separation [5], nanostructured soft/hard magnets [6], heat exchanger or cooling nanofluids [7], magneto-optical controlled wavelength filters [8], magnetorheological fluids as mechanic shock absorbers [6], up to environmental management [3,9]. Given the fact that the physical and chemical properties of MNPs largely depend on their synthesis method and chemical structure, several methods have been developed for synthesizing MNPs with different compositions [10,11,12,13].

Unprotected zerovalent iron nanoparticles (NPs) do not occur in nature because such NPs would get quickly oxidized in contact with air or water. The proper strategy to preserve the iron zerovalent nanodomains is their encapsulation in protective shells (carbon, polymers silicon, or iron oxide etc.). In comparison with conventional iron oxide NPs, it has been demonstrated that α-Fe-based NPs with the same particle dimensions exhibit a much greater magnetization and coercivity force at room temperature as the hysteresis loops revealed from magnetometry analyses [14]. Due to these properties, Fe-based NPs might be more active for generation of local hyperthermia than iron oxide NPs. Furthermore, Fe-based NPs used as MRI contrast agents have a much stronger shortening effect on T2 relaxation time than iron oxide NPs [15,16]. 

In addition, due to their high reactivity in an aqueous environment, the coated zerovalent Fe-based NPs must be studied for the characterization of certain features that are significant for biological applications, such as encapsulation, solubility, size, stability, and biocompatibility. To prevent NPs agglomeration and oxidation, which diminish their magnetism, it is important to chemically stabilize the morphology and magnetism of MNPs, during and after synthesis with non-toxic biocompatible coatings [17,18]. Several studies have focused on the design of new generation of nanoparticle systems with biocompatibility, enhanced dispersibility, internalization, and targeting capabilities improved by surface modifications [15,19]. In this respect, various biocompatible agents, such as polyethylene glycol (PEG)-derived compounds [20,21], alginate [22,23], chitosan [24], dextran [25], heparin [26], l-3,4-dihydroxyphenylalanine (l-DOPA) [27,28], phosphonates or carboxymethyldextran [29], Na carboxymethyl-cellulose (CMC-Na) [30] were used for NPs coatings. Many of those cited polymeric coating agents or other synthetic polymers, as well as the well-known biological molecules—proteins, peptides, lipids, or nucleic acids—can also form various nano-assemblies [31], without the introduction of an inorganic core. Such structures can have similar bio-applications to those of the coated nanoparticles, being useful to compare the biocompatibilities and toxicity of the two types of agents. One of the main targeted application—the drug delivery—was reviewed for the polymeric nanoparticles in [32], based on their properties for a sustained and controlled release, as well as on their small size and biocompatibility with tissues and cells. Even if they “have the potential to improve current disease therapies because of their ability to overcome multiple biological barriers and releasing a therapeutic load in the optimal dosage range”, their fate after introduction in living organisms is affected by many factors, such as composition, size, heterogeneity, surface functionality (including targeting ligands), and charge, which thus influence the biodistribution, clearance, and also their toxicity [33]. As examples for such all-organic nanoparticle-based drug delivery systems, we can cite the alginate core–Na sulfosuccinate (AOT) bilayer shell loaded with water-soluble doxorubicin, verapamil hydrochloride, clonidine hydrochloride or diclofenac sodium [34], PLGA (d,l-lactic-co-glycolic acid) nanoparticles for antimalarial artesunate agent delivery in *Plasmodium*-infected albino mice [35], or PAMAM (polyamidoamine) dendrimers as insoluble and nephrotoxic drug amphotericin B carriers [36]. However, the PAMAM dendrimer nanoparticles can also present toxicity related with the surface NH_2_ group density, which can be significantly diminished for the case of those terminating with –OH or –COOH [37]. Another type of hydrophilic organic nanoparticle used for diagnosis applications (photoacoustic contrast agent, nanoplatform for positron emission tomography (PET) or magnetic resonance imaging (MRI)) were those based on PEGylated natural melanin reported in [38]. An original approach for the synthesis of polymeric nanoparticles started from diacrylates, such as TEGDA (tetra(ethylene glycol)diacrylate), which undergo both linear polymerization and intrachain reticulation using CRP (controlled/living radical polymerization) [39]). Similar cyclized/knotted nanoparticles (composed from poly[bis(2-acryloyl) oxyethyldisulphide-co-2-(dimethylamino) ethyl methacrylate] supplementary ethylenediamine-conjugated) were successfully used as non-viral gene transfection agents for epidermal keratinocytes (skin cells) [40] based on electrostatic interactions. The same type of interactions involving positive ammonium groups of nanoparticles and negative phosphate groups from DNA (deoxyribonucleic acids), resulted in stable polyplexes that were also reported for high efficiency epidermal gene therapy, yet using biocompatible/low toxic highly branched β-aminoesters having ethylenemorpholine and hydroxypentyl groups linked to nitrogen atoms from the main polymeric chains [41,42]. Those special polymers—obtained from a triacrylate branching combined with polyethoxylated bisphenol diacrylate monomers—were tested both in vitro for biocompatibility on human cervical cancer (HeLa) cells, and in vivo on genetic collaged-defective dystrophic epidermolysis bullosa (RDEB) knockout mice, showing improved transfection efficiency and reduced cytotoxicity [43]. The synthesis of newly modified NPs for biomedical applications requires the evaluation of potential risks and toxicity effects resulting from the interaction between NPs and living cells. Failure of candidate coating can occur in a wide variety of modes, including toxicity [44], foreign body response [45] or instability in the biological milieu of hydrolytic enzymes and generation of inflammatory processes [46].

In vitro models are reliable tools to identify the effects of NPs in the human body, allowing the estimation of their toxicity and biocompatibility. Among other factors, cellular toxicity of NPs depends on the particle size, shape, porosity, surface charge, chemical composition, and colloidal stability [47]. Moreover, the nanoparticle–cell interactions and biological responses differ, depending on the cell type [48]. Many cancer cell models are used to study the biological effects of MNPs. One of them is Caco2 cell line, a human intestinal cell model which has been adopted by many researchers for exploring the toxicity of a range of NPs to the gastrointestinal system. Studies on Caco2 cell line are focused on the in vitro biocompatibility of coated magnetic iron oxide NPs for biomedical applications [49]; the effects of magnetic NPs in hyperthermia [50]; the synergistic interactions between magnetic fluid hyperthermia and the anticancer drugs [51]; the interaction with magnetic iron oxide NPs with different coatings [52]; the effect of NP size and surface charge on their interaction with cells, and the mechanism of NP internalization and toxicity [53]. The Caco2 cell line is preferred, also, because it holds many advantages due to its simplicity and reproducibility of results.

Therefore, it is important to analyze the in vitro interactions of the NPs with a specific cell line. Thus, for MNPs with potential in biomedical applications, one may then proceed towards in vivo validation and biodistribution assays. At the cellular level, the nanoparticle uptake and the mechanism by which they enter the cell has been studied, as it has important implications not only for their fate, but also for their impact on the biological systems. 

The aim of present study was to succeed the synthesis of hybrid nanoparticle aggregates containing distinct iron-based and silicon nanophases, and to prove their biocompatibility. Nanopowders were prepared by laser pyrolysis using a new special geometry for the reactive gas mixtures’ introduction. Thus, we employed a special nozzle endowed with two close and parallel tubes. Fe(CO)_5_ vapors carried by a C_2_H_4_ flow and a separate gas mixture of SiH_4_ and Ar were used as Fe and Si donors, respectively. Stabilized suspensions were prepared using synthesized NPs covered with two biocompatible agents: l-DOPA or CMC-Na, in order to evaluate their in vitro biological responses on human colorectal epithelial adenocarcinoma cells (Caco2 cell line). The effect induced in vitro was evaluated on different formulations of l-DOPA and CMC-Na-coated NPs in suspensions, and on individual components by varying the synthesis parameters focused to tailor the composition of Fe-Si nanoaggregates, as well as their magnetic properties and crystalline dimension. According to our results, the biological responses of the hybrid Fe-Si NPs were influenced by both the synthesis parameters and the type of coating/stabilizer. The results revealed that l-DOPA and CMC-Na-coated NPs display a different biological behavior on Caco2 cells, which is of great interest for further biomedical applications. 

## 2. Materials and Methods

### 2.1. Experimental Set-Up for Laser Pyrolysis

**Figure 1:**
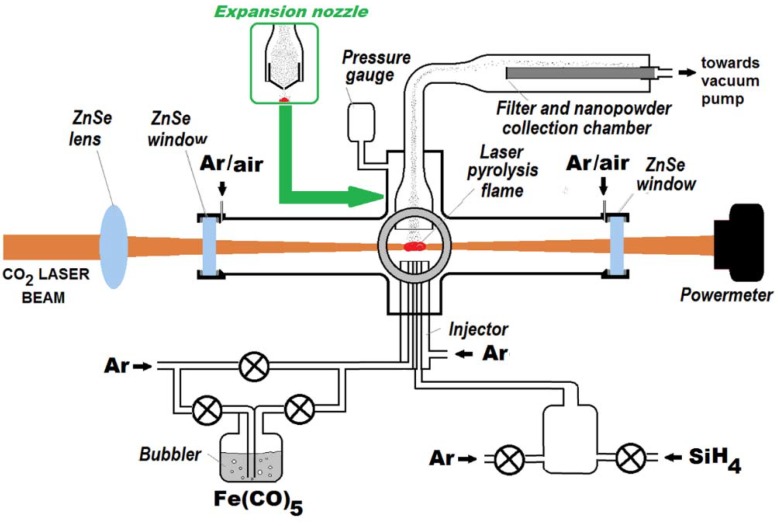
Experimental set-up used for the obtaining of nanometric Fe-Si-based hybrid nanoaggregates by laser pyrolysis followed by a post-irradiation superficial oxidation.

Distinct syntheses of Fe@Fe_x_O_y_ and Si@SiO_x_ core shell nanopowders were already performed using previously described laser pyrolysis protocols [14,54,55]. Briefly, for producing nanosized iron or silicon nanopowders in the typical procedure, the focused continuous wave or pulsed CO_2_ laser radiation (λ = 10.6 µm) orthogonally crossed the gas flows, emerging through the central inlet tube dedicated for reactive gas mixture: (1) C_2_H_4_ and Fe(CO)_5_ vapors for Fe based nanopowders, or (2) SiH_4_ in combination with an inert gas or C_2_H_4_ for Si-based nanopowders. The confinement of gas precursors toward the flow axis and of the freshly nucleated particles was achieved by a coaxial argon (or other inert gas) flow. Here, we employed a COHERENT Diamond E-400 (Coherent, Santa Clara, CA, USA) CO_2_ pulsed laser emitting in infrared domain at 10.6 µm with 90 kHz frequency, for the simultaneous synthesis of hybrid nanomaterials with distinct Fe-based and Si nanocrystals, just by approaching the two central gas flows via a nozzle with two parallel nearby inner tubes dedicated for Fe and Si precursors, respectively. In order to control the aggregation process, two different geometries for the reaction product evacuation have been used: (1) a classical one with a cylindrical geometry having a 3 cm^2^ cross section placed at 3 mm above the reaction zone, or (2) a tronconical one (narrow escape, similar with the expansion nozzle used in [56]), where the smaller base-endowed with a 0.05 cm^2^ hole—was placed at 1.5 mm above the reaction zone, as presented in Figure 1. The second exhaust trajectory generated a quick quenching for the exhaust products (powder and resulted gaseous components). In all experiments (labeled Fe-Si), the Fe(CO)_5_ (the iron precursor) vapors entrained by ethylene emerged through the first central inlet tubes, while a mixture containing SiH_4_ and Ar emerged into the reaction chamber through the neighboring second central inlet (see Figure 1). The Fe and Si precursors, and partially, the ethylene, were allowed to dissociate in two distinct but adjacent flames, due to the resonant interactions between the laser beam and both ethylene and silane (having thus the role of laser energy transfer agents or sensitizers) and they transferred the resulted heat via intermolecular collisions to both (Fe-rich and Si-rich, respectively) reactive mixtures. 

The most relevant samples selected for this study were synthesized using the parameters presented in Table 1. In all cases, an annular external argon flow (2500 sccm) assured the confinement toward the central flow axes. At the end of synthesis, the input gas streams were stopped, the remaining gas extracted with the vacuum pump from both reaction and collection chambers, followed by synthetic air (the oxidizer agent) slowly being introduced in the main chamber through the lateral outlets (located near the IR-transparent windows). This “soft oxidation” treatment ensured the development of an oxidic shell at zerovalent iron surfaces, preventing thus the particles burning upon air exposure [57]. A similar oxidic shell (made from SiO_2_) seems also to superficially cover the Si nanoparticles due this treatment.

After the synthesis and superficial oxidation treatment, the morphology and composition of the as prepared NPs were characterized by X-ray diffraction (XRD), energy-dispersive X-ray analysis (EDX), transmission electron microscopy (TEM), and selected area electron diffraction (SAED). Also, their magnetic features were investigated via vibrating sample magnetometry (VSM). Hybrid nanoaggregates might contain two distinct components: a magnetic one due to presence of Fe-based nanodomains, and a non-magnetic one if Si rich nanomaterial co-occur randomly mixed with the first one in the same powder. This fact appears predominantly, when the two parallel inlets are positioned too far apart (more than 2 mm). For this reason, only those samples that separate magnetically more than 95 wt % from a methanol-based suspension were used in the next studies. Also, all three samples selected here (see Table 1) were synthesized using 1.0 mm distance between the two inlet tubes, and they revealed this macroscopic magnetic homogeneity.

### 2.2. Synthesis and Coating of Hybrid Fe-Si Nanoparticles

As raw materials for the laser pyrolysis synthesis, we employed iron pentacarbonyl (97%) from Sigma-Aldrich (St. Louis, MO, USA) as vapors from volatile liquid and gaseous ethylene (99.999 vol. %), silane (99.99% vol. %), argon (99.9999 vol. %), and synthetic air (having 20% O_2_ and 80% N_2_ vol. %) from Linde Gas (Timisoara, Romania). For the water-based nanoparticle suspensions’ preparation, we employed l-DOPA (3,4 dihydroxy-l-phenylalanine, product code D9628), carboxymethylcellulose sodium salt (low viscosity, product code C5678) and Dulbecco’s phosphate buffered saline (PBS, product code D1408), all purchased from Sigma-Aldrich (St. Louis, MO, USA).

The preparation of stabilized suspensions was done according the same procedure as in [27] when l-DOPA was used as stabilizer. As a particularity, Fe-Si nanoaggregates showed a good stability in distilled water (pH ~5.5, due to the atmospheric CO_2_ absorption) but their magnetic properties decreased down to 60% in comparison with those dispersed in acetone (a base fluid presumed to be inert, which does not modify the particles magnetic properties). Due to this reason, the homogenized dry mixture of nanopowder and CMC-Na stabilizer was slowly introduced in water under the combined action of the ultrasonic horn disperser (20 mm diameter, Hielscher UIP 1000-hd Ultrasonic Homogenizer, Hielscher Ultrasonics, Teltow, Germany) and a vibrating thin rod during 5 min, and then for the entire suspension, the ultrasonic treatment was continued for 30 min using an external cold water bath, to maintain a temperature around 30 °C. For catechol stabilization, firstly, a 3 g/L l-DOPA hot solution was prepared, then the magnetic powder was added using the vibrating rod and the ultrasonic bath (Labsonic LBS2 from Falc Instruments, Treviglio, Italy), sealed and ultrasonicated on the same bath for 5 h at 70 °C, followed by the same half hour horn ultrasonication, also at 70 °C temperature. Using these procedures, the magnetic saturation of final stabilized suspension dropped only by 15% in comparison with the one made at the same powder concentration, in acetone. In both cases, the nanopowders were suspended and stabilized in distilled water, and then transferred in PBS.

### 2.3. Characterization of Hybrid Fe-Si Nanoparticles and Resulted Suspensions

The crystalline phase composition of raw nanopowders was evaluated using a PANalyticalX’Pert MPD theta–theta X-ray diffraction (XRD) apparatus (PANalytical, Almelo, Netherlands) using a Cu K-α source (0.15418 nm), while their morphology and structure were examined with a Philips CM 120ST (120 kV) Transmission Electron Microscope (TEM), (Philips, Amsterdam, Netherlands). Energy-dispersive X-ray spectroscopy (EDS) measurements for elemental analysis were performed inside a scanning electron microscope FEI Quanta Inspect S (FEI, Hillsboro, OR, USA) at 10 kV accelerating voltage, using an ELEMENT Silicon Drift Detector from EDAX Co (FEI, Hillsboro, OR, USA). Magnetic hysteresis curves were obtained at room temperature using a vibrating sample magnetometer (VSM) under applied fields up to 1000 kA/m. A Malvern Zetasizer apparatus (Malvern, Worcestershire, UK) was employed for the aqueous media suspended particles mean hydrodynamic size analyses using dynamic light scattering (DLS) method.

### 2.4. Cell Line Culture and Treatment

The Caco2 cell line, originally derived from a human colorectal adenocarcinoma, was purchased from American Type Culture Collection (ATCC HTB-37, ATCC/LGC Standards GmbH, Wesel, Germany). Human Caco2 cell line is a continuous line of heterogeneous epithelial colorectal adenocarcinoma cells, rapid and easy to culture, and is also used to predict in vivo toxicity [58]. Caco2 cells are approximately 30 to 70 μm, spindle- or polygon-shaped (high cell density), with adherent cells growing as a confluent monolayer.

The cell cultures were grown in 75 cm^2^ flasks in complete Minimum Essential Medium (MEM; M0643, Sigma-Aldrich, St. Louis, MO, USA) supplemented with 1% antibiotic–antimycotic mix solution (A5955; Sigma-Aldrich, St. Louis, MO, USA) and 20% fetal bovine serum (10270-106, origin South America, Gibco, by Life Technologies, Carlsbad, CA, USA) and maintained at 37 °C in humidified atmosphere (95%) with 5% CO_2_. 

To evaluate the biological effect induced in Caco2 cells by hybrid Fe-Si NPs exposure, the cells were seeded in 96 and 24-well plates at a density of 5 × 10^4^ cells/mL and allowed to attach overnight. Then, hybrid Fe-Si NPs and the two stabilizers (l-DOPA and CMC-NA) were serially diluted (0 = control, 12.5, 25, 50, 100, 200 µg/mL for NPs, and 7.5, 15, 30, 60, 120 µg/mL for corresponding concentrations of stabilizers in NPs suspensions) in cell culture medium, and incubated with the cells for 24 and 72 h. 

### 2.5. Cell Viability and IC_50_ Evaluation

Cell viability was measured after cell exposure to nanoparticles by 3-(4,5-dimethylthiazol-2-yl)-2,5-diphenyltetrazolium bromide (MTT) spectrophotometric test [59]. Briefly, the cells were seeded in 96-well plates at a density of 10^4^ cells/well/200 μL, and allowed to adhere for 24 h. Hybrid Fe-Si NPs (0–200 μg/mL) and the two stabilizers (0–120 μg/mL) were added into culture media and incubated for 24 and 72 h. After each time interval, the medium with NPs was removed from all wells, and 75 μL of 1 mg/mL MTT solution (M2128, Sigma-Aldrich) was added for 2 h at 37 °C. The formazan produced by MTT reduction in metabolic active cells was solubilized in 150 μL isopropanol, and the absorbance was measured at 595 nm using a FlexStation 3 microplate reader (Molecular Devices, San Jose, CA, USA). 

IC_50_ values or inhibitory concentrations were calculated to identify the dose that inhibits 50% of the viability of Caco2 cells. For calculating the IC_50_ values, the treatment concentrations (x) and the viability inhibition rate obtained by the MTT (y) test were used. The data were plotted by linear regression, and the IC_50_ values were estimated using the equation: Y = a × X + b; IC_50_ = (0.5 − b)/a.

### 2.6. Cell Morphology and F-Actin Cytoskeleton Imaging

Morphologic characteristics of Caco2 cells after exposure to NPs were analyzed by optical and fluorescence microscopy. Bright-field representative images with Caco2 cells were acquired by phase contrast microscopy using an inverted microscope Olympus IX73 (Olympus, Tokyo, Japan) and CellSens Dimension imaging software (ver. 1.11, Olympus, Tokyo, Japan). Cell actin cytoskeleton was fluorescently labeled using Alexa Fluor 488 phalloidin dye (A12379, Molecular Probes by Life Technologies, Carlsbad, CA, USA), which binds F-actin filaments with high selectivity, providing a green fluorescence. Briefly, after NPs exposure cells were fixed with 4% paraformaldehyde for 10 min and permeabilized with a solution of 0.1% Triton X-100 in 2% bovine serum albumin for 45 min. Finally, cells were stained for 45 min at room temperature with 150 nM Alexa Fluor 488 phalloidin solution. Images were captured with an inverted fluorescence microscope Olympus IX71 (Olympus, Tokyo, Japan) with Cell^F software (ver. 5.0, Olympus, Tokyo, Japan) using identical settings. Fluorescence intensity of phalloidin-positive cells was quantified by using ImageJ (ver. 1.47q, Bethesda, MD, USA), and the results expressed as background-corrected integrated fluorescence density. Six images for each sample were analyzed and the fluorescence intensity was measured. The graph was represented using the mean fluorescence ± standard deviation.

### 2.7. Lactate Dehydrogenase (LDH) Assay

The LDH amount released in culture medium was assessed as a measure of cell membrane integrity using the In Vitro Toxicology Assay Kit, Lactic Dehydrogenase-Based (TOX7, Sigma-Aldrich, St. Louis, MO, USA). According to the manufacturer’s instructions, volumes of 50 µL of culture supernatants removed from the same 96-well plates used for MTT test, were incubated with 100 µL mix composed from equal parts (1:1:1) of dye, substrate, and cofactor for 30 min in dark. The reaction was stopped by adding 15 µL of 1 N HCl, and the absorbance was read at 490 nm using a microplate reader.

### 2.8. Measurement of ROS Production

The level of intracellular reactive oxygen species (ROS) generated after exposure to hybrid Fe-Si NPs in Caco2 cells was measured as previously described [60] using 2′,7′-dichlorofluorescein diacetate (H2DCF-DA; Sigma-Aldrich, St. Louis, MO, USA). Only two NPs concentrations were investigated: 25 and 50 µg/mL, selected after cell viability evaluation. The cells were seeded in 96-well plates at a density of 2 × 10^4^ cells/well and after 1 to 4 h of incubation with NPs incubation, ROS production was recorded (as relative fluorescence units, RFU) using a fluorescence microplate reader FlexStation 3 (Molecular Devices, , San Jose, CA, USA) at 485 nm ex./520 nm em. wavelength.

### 2.9. Protein Extraction

Caco2 cells, harvested from culture flasks, were washed with PBS, trypsinized, and centrifuged at 1500 rpm for 5 min. Cell pellets were washed again, resuspended in 400 µL of PBS, and then sonicated on ice three times, for 30 s each. The total protein extract was centrifuged at 3000 rpm for 10 min at 4 °C. Aliquots of the supernatant were used for further determination. The protein concentration, expressed as mg/mL, was measured by Bradford method, using bovine serum albumin as standard [61].

### 2.10. Quantification of GSH Content

The intracellular reduced glutathione (GSH) concentration was evaluated using the Glutathione Assay Kit (CS0260, Sigma-Aldrich, St. Louis, MO, USA). Briefly, after treatment with NPs, protein extracts were precipitated with 5-sulfosalicylic acid (1:1) and the supernatants were recovered. A volume of 10 µL from each sample was added in 150 µL assay buffer containing 5,5′-dithiobis(2-nitrobenzoic acid) (DTNB; D8130, Sigma-Aldrich, St. Louis, MO, USA), and incubated for 10 min at room temperature. The 5-thio-2-nitrobenozoic acid (TNB) formed was measured spectrophotometrically at 405 nm. A calibration curve (3.125–200 µM) was similarly prepared using glutathione solution as standard. The results were expressed in nmoles GSH/mg of protein and represented as a ratio of treated cells compared to untreated cells.

### 2.11. Immunoblotting of Nuclear Factor E2-Related Factor 2 (Nrf-2) 

Equal amounts of protein (30 µg) from treated and untreated Caco2 cells were electrophoresed through an SDS-polyacrylamide gel (10% resolving gel) in Tris-glycine buffer at 90 V for 2 h. The proteins were transferred from the SDS-polyacrylamide gel to Immun-Blot PVDF membrane (Bio-Rad, Hercules, CA, USA) using a wet transfer system. Membranes were developed using WesternBreeze Chromogenic Anti-Rabbit Kit (WB7105, Invitrogen, Carlsbad, CA, USA) and rabbit polyclonal primary anti-Nrf-2 antibody (sc-722, Santa Cruz Biotechnology, Heidelberg, Germany) according to the manufacturer’s instructions. The protein bands were detected by staining with the BCIP/NBT substrate and visualized with the ChemiDoc Imaging System (Bio-Rad, Hercules, CA, USA). Quantification of bands on Western images was done using the Image Lab software (ver. 5.2, Bio-Rad, Hercules, CA, USA). To normalize Nrf-2 levels, Nrf-2 band intensity was divided by β-actin (A1978, Sigma-Aldrich, St. Louis, MO, USA) band intensity (which serves as reference protein), and represented as percentages of control.

### 2.12. Statistical Analysis

All tests were performed in triplicate, and data were shown as mean ± standard deviation (SD). The statistical Student’s *t*-test was performed for biological tests to analyze significant differences when comparing treated cells with controls. A value of *p* < 0.05 was considered significant.

## 3. Results and Discussion

### 3.1. Hybrid Fe-Si Nanoparticles Preparation and Characterization

**Figure 2:**
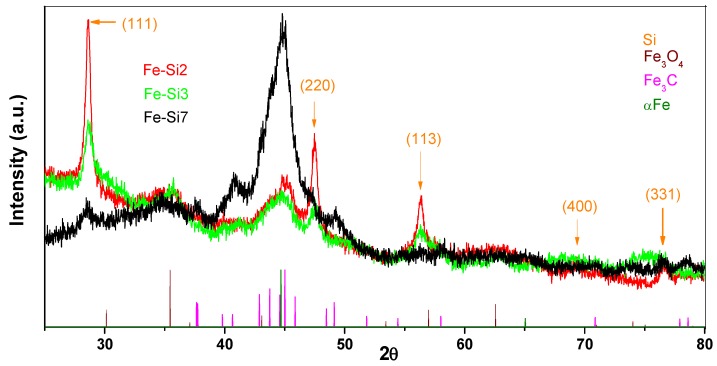
Superposed X-ray diffractograms for the Fe-Si2, Fe-Si3, and Fe-Si7 raw nanopowders, and the reference diffractograms for Fe-based phases.

In the Figure 2, superposed X ray diffractograms of the nanopowders from three selected experiments (two employing large cylindrical gas/particles evacuation geometry—Fe-Si2 and Fe-Si3, and one using the expansion nozzle—Fe-Si7) are presented. The elemental silicon phase can be observed for all powders, but for those synthesized in classical cylindrical geometry, the corresponding peaks are more intense, especially for the Fe-Si2 sample where the highest silane flow was used. Highest Si atomic concentration was found also in the Fe-Si2 sample (see Table 2), confirming the tendency of decreasing the amount of silicon detected in the resulted nanopowders with diminishing SiH_4_ flow. The mean silicon crystallite sizes in the nanopowders obtained in this geometry decreased with the diminishing of SiH_4_ flow, as can be calculated using Debye–Scherrer formula (17.5 nm for Fe-Si2 vs. 13.3 nm for Fe-Si3). The presence of Fe-based phases, formed by iron pentacarbonyl decomposition under ethylene sensitizer influence, is proved by the identification of Fe_3_C (cohenite) peaks, the most intense being those at 2θ = 45°. Other phases, such as Fe_3_C_7_ and αFe, can also be present in small quantities, given that most of their peaks overlap with those of the Fe_3_C phase. Even if we used the same ethylene-entrained Fe(CO)_5_ vapor flow in all three experiments, it seems that the expansion nozzle favorizes their decomposition. Thus, the Fe-Si7 powder exhibits the most intense peak at 2θ = 45°, accompanied by smaller peaks at 41° and 49°, all attributed to Fe_3_C phase, formed by the ethylene decomposition on the freshly-formed iron clusters resulting from Fe(CO)_5_ decarbonylation and coalescence, followed by the solubilization of the resulting carbon species. This fact is confirmed by the EDS measurements (see Table 2) which show that the Fe-Si_7_ samples have the highest iron atomic concentration. A lower intensity peak can be observed for all three samples at 2θ ~36°, could be attributed to γFe_2_O_3_/Fe_3_O_4_ phase, formed by superficial oxidation of iron-based phases during the post-synthesis passivation process.

**Figure 3:**
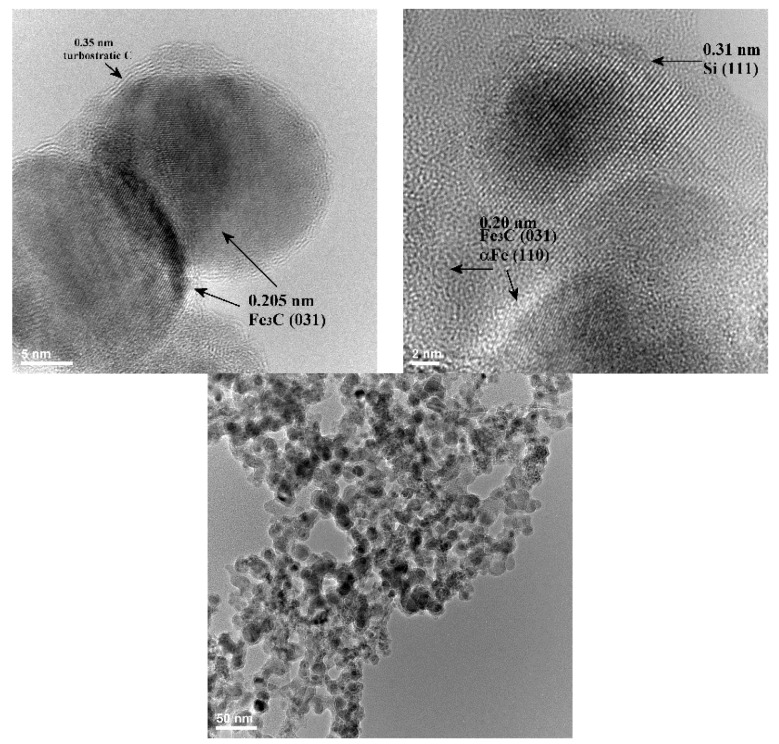
Higher definition (left: Fe-Si3, center: Fe-Si7 samples) and lower definition (right: Fe-Si7 sample) TEM images of nanoparticles from as-synthesized powders.

Figure 3 contains a series of transmission electron microscopy images from Fe-Si3 and Fe-Si7 samples, showing the round morphology of the observed nanometric particles. The left high-resolution image shows two nanoparticles with diameters around 22–25 nm from the Fe-Si3 powder, where the (031) cohenite crystalline planes—distanced at 2.05 Å—were identified. These nanoparticles are covered with a thin layer of turbostratic (composed from 3 or 4 stacked graphenes distanced at ~3.5 Å) mixed with amorphous carbon. This carbon-based layer can be formed by excess carbon precipitation from the carbon-saturated iron-based NPs upon cooling after the reaction zone. The central high resolution transmission electron microscopy (HR-TEM) image (from Fe-Si7 sample, synthesized with the expansion nozzle) shows a few aggregated nanoparticles deposed onto the amorphous carbon membrane which covers the electron microscope grid. In the upper one, (100) parallel crystalline planes, distanced at 0.31 nm, belonging to silicon phase, can be clearly seen, which extend over 12 nm. In the lower part of same image, another two smaller crystalline zones can be identified, with 0.20 nm interplanar distance, which could be attributed to (031) planes of Fe_3_C or to (110) planes of αFe. The third TEM image from the right part of Figure 3 shows the extended fractal aggregates of small (3–5 nm) NPs from the same powder, Fe-Si7. Even at this lower magnification, the core–shell structure of the chained nanoparticles can be clearly noticed.

Table 3 summarizes some of the magnetic parameters of the raw nanopowders, extracted from the room temperature hysterezis magnetization curves. As expected, the highest magnetization (47 emu/g) can be found for the Fe-Si7 sample with the higher Fe and lower Si content. The low values of the coercivity reflect the near superparamagnetic behavior of the NPs, in concordance with their dimensions. The maximum permeability values are of the same size order with those having of Fe-based core and ZnO shells synthesized also by one-step laser pyrolysis, and recently reported by us [36].

### 3.2. Coating of Hybrid Fe-Si NPs with l-DOPA or CMC-Na

**Figure 4:**
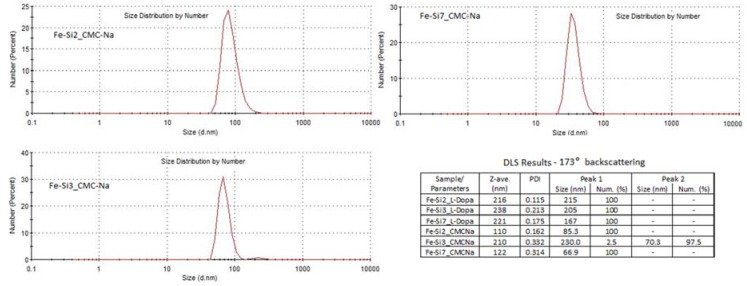
DLS size distribution diagrams of aqueous suspensions containing Fe-Si nanopowders stabilized with CMC-Na and inserted tabulated values of mean hydrodynamic sizes of water-suspended Fe-Si nanoparticle aggregates stabilized with CMC-Na and with l-DOPA.

The dimensional distribution (by number) of polyanionic CMC-Na polyelectrolyte-coated nanoparticle aggregates from the Fe-Si samples is presented in Figure 4. A monomodal asymmetric distribution for Fe-Si2 NPs polyelectrolyte-coated in suspension can be observed, with the mean hydrodynamic size centered at 85.3 nm. However, the scattering intensity counted DLS size distribution from the same suspension (not shown here) reveals a bimodal distribution, which reflects the presence of two distinct populations of aggregates, the smaller having sizes centered around 81 nm, whereas the bigger ones are slightly more than three time larger (centered ~264 nm). Corroborating those two DLS size distributions, one can conclude that in the CMC-Na-stabilized Fe-Si2 NPs suspension the number of bigger aggregates is very low. The suspension from Fe-Si3 NPs hydrophilized with CMC-Na shows a bimodal distribution (by number), where the smaller nanoparticle aggregates (~70 nm) far outnumber the bigger ones (~230 nm), a situation similar with the previously discussed NPs dispersion. The Fe-Si7 NPs suspended with CMC-Na shows also an asymmetric monomodal size distribution with a reduced “tail” in the 70–90 nm zone, the mean size in this case, being around 67 nm. If we compare the DLS size distributions for all, synthesized suspensions, we can conclude that the CMC-Na agent induces the formation aggregates having smaller sizes than those induced by l-DOPA, and that the use of the expansion nozzle (Fe-Si7 sample) favored also smaller aggregates. 

Since both superficial silicon dioxide and iron oxide formed by passivation of Fe-Si NPs have a hydrophilic behavior, in neutral media they tend to aggregate via hydrogen bonds formation between hydroxyl surface groups. It seems that their coating with CMC-Na polyanionic chains limits the aggregation, and also stabilizes the small aggregates against further coalescence and sedimentation by electrostatic repulsions between negatively charged carboxyl groups. 

**Figure 5:**
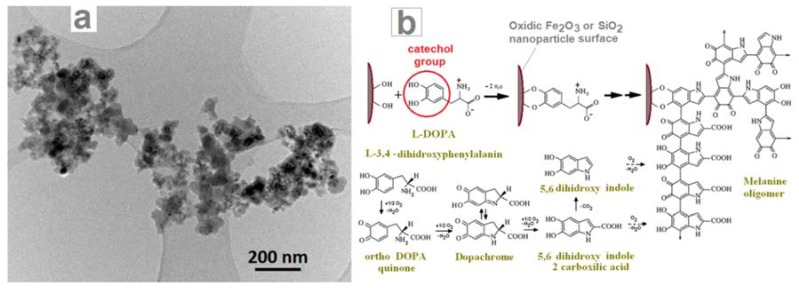
(**a**) TEM image of l-DOPA coated aggregates of nanoparticle derived from Fe-Si3 sample; (**b**) Proposed mechanism for l-DOPA attachment and polymerization to oligomelanin on the nanoparticles oxidic surface.

The successful ultrasonication-assisted l-DOPA coating of laser pyrolysis-synthesized iron oxide nanoparticles in aqueous media was reported by us in [27,62], where a supplementary rhodamine derivate dye labelling was achieved. During this coating procedure, a partial oxidative polymerization of l-DOPA (under the action of dissolved oxygen) to oligomelanins seems to occur, as proven by the darkening of colorless pure l-DOPA solution at the same concentration, which was subjected to an identical sonication treatment. The presence of a conformal coating (~15–20 nm thickness), which also embeds multiple nanoparticles from the Fe-Si3 sample, can be observed in the TEM image from Figure 5a. The chemical anchoring of l-DOPA molecules to the oxidic surfaces of Fe-Si type, in a similar way to that reported for magnetite [63] nanoparticles, occurs via the orthophenolate (catechol) part—see Figure 5b, while the remaining zwitterionic amino acid part assures a high hydrophilicity and electrostatic stabilization at neutral pH. Similar stabilization can also be induced by indole and/or carboxy groups from oligomeric melanins (related with natural eumelanins) resulted by l-DOPA sonication-induced oxidative polymerization as schematically shown in Figure 5b, according to a mechanism also proposed in [64].

### 3.3. Biocompatibility Assessment

#### 3.3.1. Cytotoxicity 

The new materials developed for biomedical applications require the simultaneous fulfillment of three major conditions: biocompatibility, low toxicity, and biodegradability. Various nanostructures based on iron and silicon have been reported to be highly biocompatible [65,66,67], and easily metabolized by the body as orthosilicic acid Si(OH)_4_ or iron ions. The retention of NPs in the body primarily can negatively affect organs, including liver, kidneys, stomach, and intestines. 

**Figure 6:**
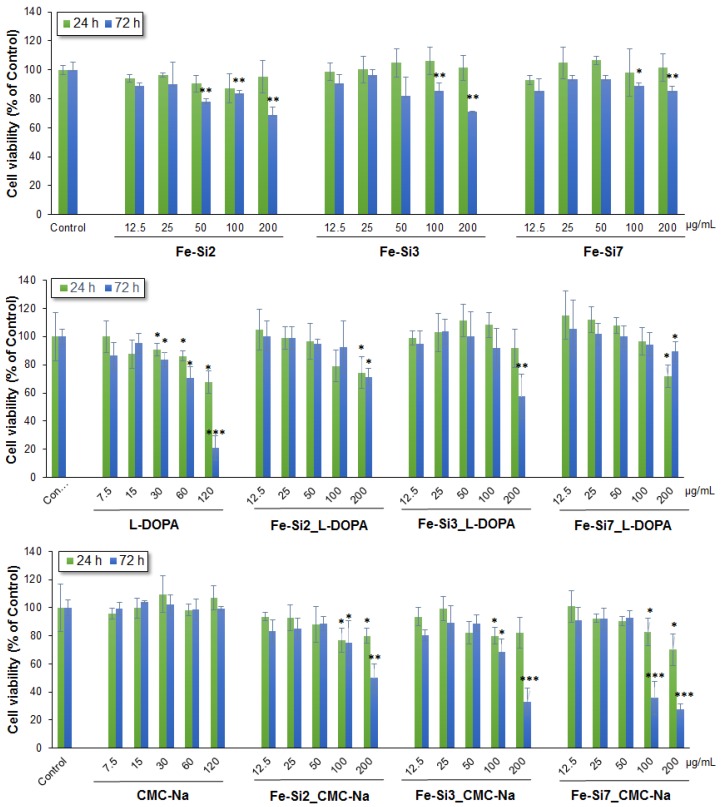
Cell viability of Caco2 cells after 24 and 72 h exposure to different doses (0–200 µg/mL) of hybrid Fe-Si NPs alone and stabilized with l-DOPA and CMC-Na (0–120 µg/mL). Data are expressed as the mean ± SD (*n* = 3) and represented as percentages of untreated control (100% viability). * *p* < 0.05; ** *p* < 0.01; *** *p* < 0.001 versus untreated control.

To assess the biocompatibility of hybrid Fe-Si NPs coated with l-DOPA and CMC-Na, doses ranging from 0–200 µg/mL were exposed to Caco2 cells for 24 and 72 h. Untreated cells and cells treated with the individual components of NPs were used as controls. The results indicated no significant change in Caco2 cell viability exposed to concentrations between 0–100 μg/mL l-DOPA-coated Fe-Si NPs and 0–50 μg/mL CMC-Na-coated Fe-Si hybrid NPs (Figure 6). At the highest dose of 200 μg/mL, NP cell viability decreased after 72 h exposure by 29%, 43%, and 11% in the presence of Fe-Si2_l-DOPA NPs, Fe-Si3_l-DOPA NPs, and, respectively, Fe-Si7_l-DOPA NPs. By comparison, Fe-Si7_CMC-Na NPs (200 μg/mL) were the most toxic, causing a decrease by 73% of cell viability after 72 h of exposure, compared to untreated control. By comparison, the decrease of viability in the Caco2 cells exposed to uncoated NPs was lower, the maximum decrease was induced by 200 μg/mL Fe-Si NPs after 72 h. Surprisingly, the most toxic component for Caco2 cells was l-DOPA which has an IC_50_ value of 81.2 μg/mL. However, the IC_50_ values for the l-DOPA stabilized NPs were higher compared with those stabilized with CMC-Na (Table 4).

By now, l-DOPA and CMC-Na have been used to stabilize various NPs, including iron oxide [68], magnetic Fe-MWCNT [69], Fe@C [70], zinc oxide (ZnO) [71], silica (SiO_2_), and titania (TiO_2_) ones [72], as well as anticancer drug carriers [73] etc., but the interactions with cells and tissues were not entirely described and understood.

l-DOPA is an amino acid, precursor of dopamine, biosynthesized naturally by a number of plants and animals. In humans, it is obtained through the metabolic pathway of catecholamines, being biosynthesized directly from l-tyrosine. Some in vitro studies have reported toxic effects of l-DOPA [74,75], whereas others have demonstrated a protective action, similar to the beneficial effects of some dopamine receptor agonists. Furthermore, the results of many in vivo experiments, as well as of clinical trials, did not demonstrate l-DOPA toxicity or remained inconclusive [76]. On the other hand CMC-Na “generally recognized as safe” (GRAS) by the US Food and Drug Administration is widely used in diverse industries including medical applications. The in vitro and in vivo biocompatibility CMC has been demonstrated [22,77] as well as limited biodegradation by glucose residues releasing [78].

In our case, as resulted from Figure 6, NPs coated with CMC-Na presented a higher toxicity compared with those coated with l-DOPA at high doses in Caco2 cells.

**Figure 7:**
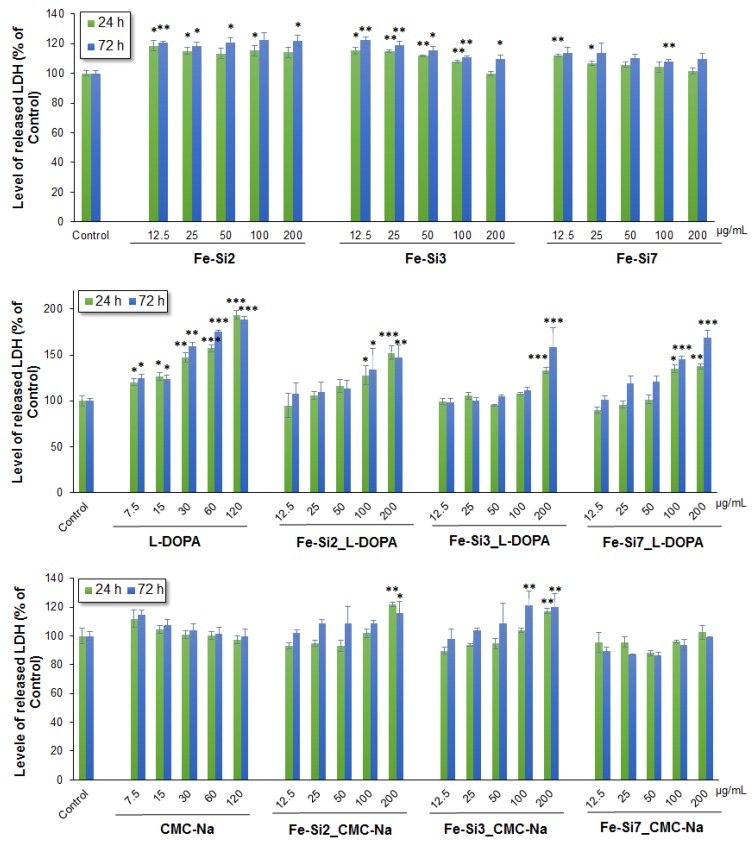
Level of lactate dehydrogenase (LDH) released from Caco2 cells after 24 and 72 h exposure to different doses (0–200 µg/mL) of hybrid Fe-Si NPs alone and stabilized with l-DOPA and CMC-Na (0–120 µg/mL). Data are expressed as the mean ± SD (*n* = 3) and represented as percentages of untreated control (100% viability). * *p* < 0.05; ** *p* < 0.01; *** *p* < 0.001 versus untreated control.

The release of lactate dehydrogenase (LDH) into the surrounding culture medium was assessed as a marker for membrane integrity and necrotic events. Upon incubation with all tested suspensions, high activity of LDH was found in the medium of Caco2 cells exposed to stabilized NPs and to l-DOPA (Figure 7). A lower increase of LDH level was also noticed after incubation of Caco2 cells with Fe-Si2, Fe-Si3, and Fe-Si7 NPs compared to untreated control, whereas no release of LDH was observed after cell exposure to Fe-Si7_CMC-Na NPs and CMC-Na alone. These data were in accordance with the results of MTT test and suggest that stabilization of hybrid Fe-Si NPs with l-DOPA and CMC-Na could influence the interaction with Caco2 cells and increases their toxicity at higher doses.

#### 3.3.2. Cell Morphology and Dispersion of NPs in Culture

**Figure 8:**
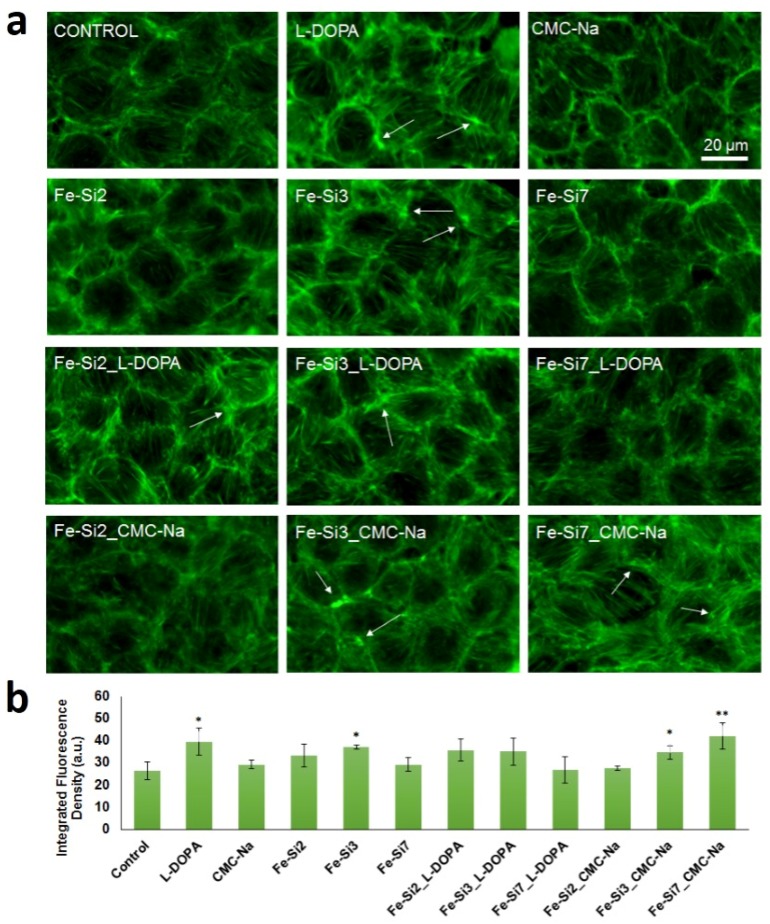
F-actin cytoskeleton in Caco2 cells after exposure for 72 h to 50 µg/mL hybrid Fe-Si NPs. (**a**) Direct immunofluorescence of actin staining by Alexa Fluor 488 phalloidin (green fluorescence). White arrows indicate F-actin aggregation and actin bundles accumulation on cell periphery Magnification: 40×. Control represents untreated cells. Scale bar = 20 µm; (**b**) Quantification of F-actin fluorescence.

To study the morphology of treated Caco2 cells, two doses of 25 and 50 µg/mL of Fe-Si NPs were chosen based on cytotoxicity test outcomes. We inspected the filamentous actin cytoskeleton of Fe-Si NP-treated Caco2 cells as a measure of preservation of the overall cellular architecture. Untreated cells showed a regular structure made up of aligned and tightly compacted F-actin or actin bundles (Figure 8a). After incubation of Caco2 cells with each type of NP, no obvious changes in the cortical F-actin system were caused by doses of 25 μg/mL (data not shown). The corresponding morphologic structures remained detectable underneath the plasma membranes of most cells. However, some alterations were noticed in cells exposed for 72 h to 30 µg/mL l-DOPA and 50 µg/mL Fe-Si3, Fe-Si3_CMC-Na, and Fe-Si7_CMC-Na (Figure 8b). Formation of some F-actin aggregates and/or inclusion bodies and a great proportion of actin bundles accumulated on cell periphery were noticed (Figure 8a). By contrast, F-actin cytoskeleton was largely reserved after incubation with Fe-Si7 and Fe-Si7_l-DOPA samples. As stated above, we show that cell viability was not compromised by the presence of the F-actin alterations.

**Figure 9:**
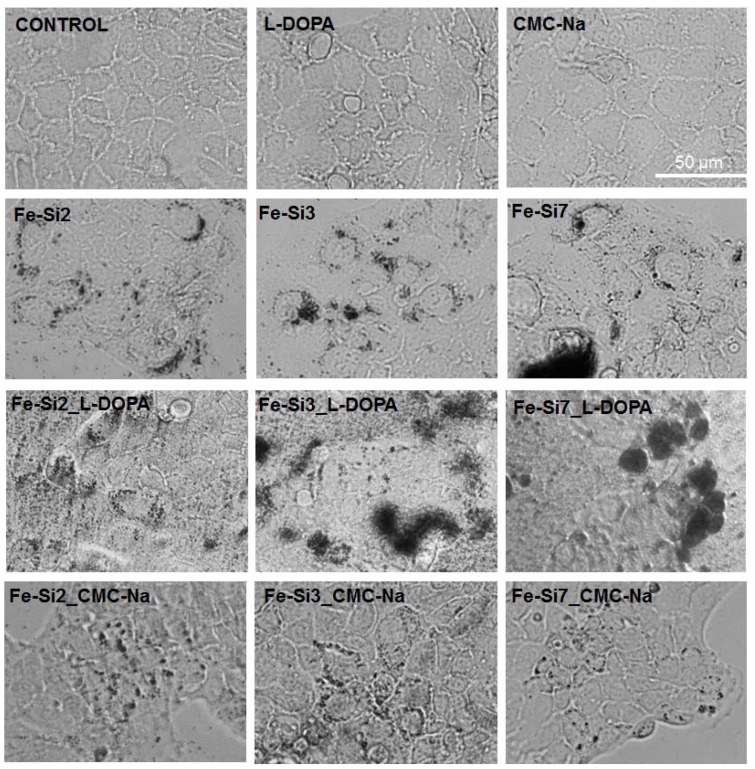
Images representing Caco2 cell morphology after exposure for 72 h to 50 µg/mL hybrid Fe-Si NPs. Bright-field images showing NP dispersion and internalization into the cells. Data are representative of 3 separate experiments with 3 replicates for each experimental condition. Control represents untreated cells. Scale bar = 50 µm.

The degree of internalization was different between Fe-Si NPs samples. Unstabilized Fe-Si NPs presented a low dispersion in the aqueous suspension, as well as in the culture medium, which hindered the internalization on cellular level. The large aggregates of NPs are impossible to penetrate the cellular membrane, and so the interaction with the cells remained, for the most part, limited to the outer membrane. All stabilized suspensions of NPs were initially well-dispersed solutions, but after their addition in the culture medium, l-DOPA -coated NPs have formed large aggregates, especially the Fe-Si7_l-DOPA sample (Figure 9), probably due to the presence of ionized carboxyl and amino groups that could interact electrochemically between individual NPs and between NPs and amino acids and proteins from the culture media. Figure 9 shows the dispersion of hybrid Fe-Si NPs in the cell culture medium, and their internalization in the Caco2 cell cytoplasm. The Fe-Si NPs stabilized with CMC-Na presented a better dispersion in medium compared with those stabilized with l-DOPA. As it can be seen, no internalization was observed for Fe-Si7_l-DOPA sample.

In a culture medium environment, rich in serum proteins, NPs are covered by the so-called protein corona, which is influenced by the surface properties of the coated NPs in terms of type and amount of absorbed proteins. This protein corona plays an important role in their interaction with the cells and tissues, and thus in biological responses, therapeutic efficiency, and toxicity of NPs [79].

l-DOPA and CMC-Na are chemically different. In the culture medium (pH 7.4), l-DOPA has a neutral charge. According to several protein corona studies, neutral surfaces are prone to adsorb high amount of serum proteins, which can result in higher blood circulation time in vivo [80,81]. Carboxylic acid groups can act as anchor points for addition of secondary molecules by covalent bonding with amine groups, e.g., via carbodiimide chemistry [80]. 

On the contrary, CMC sodium salt is an anionic derivative of cellulose, negatively charged at neutral pH (pKa of 4.3), in which the hydroxyl groups are partially or fully substituted by carboxymethyl groups (–CH_2_–COOH). Due to the ionic nature of CMC-Na, it can interact with proteins to form soluble and stable complexes. Also, the polar groups of CMC-Na (–OH, –COOH) can react with metal ions (Fe^2+^, Fe^3+^, Ca^2+^, Mg^2+^) by electrostatic forces.

The formation of aggregates in cell culture medium favors the absorption of more serum protein onto the NP surface, so the interaction of NPs with cell membrane is quite limited. 

In the case of Fe-Si7_l-DOPA sample, the interaction occurred most likely at the cell membrane surface, thus explaining the increased level of LDH, starting with 24 h of incubation with 100 and 200 µg/mL doses. Also, the NP uptake was low. On the contrary, CMC-Na NPs (Fe-Si7_CMC-Na sample) presented a high dispersion in the culture medium and a high internalization in Caco2 cellular cytoplasm occurred (Figure 9). Similar changes were registered for the other CMC-Na coated NP samples. This could indicate that mechanism of toxicity is influenced by the type of coating and degree of dispersion in the culture medium. 

#### 3.3.3. Oxidative Stress

The generation of reactive oxygen species (ROS) in Caco2 cells was analyzed up to 4 h of exposure to 25 and 50 µg/mL hybrid Fe-Si NPs. As shown in Table 5, the production of ROS was time-dependent. The unstabilized Fe-Si NPs generated the highest amount of ROS. All samples CMC-Na-coated Fe-Si NPs induced ROS post-exposure at both doses of 25 and 50 µg/mL. In the case of l-DOPA-coated Fe-Si NPs, higher ROS levels were obtained for 25 µg/mL dose. No significant increase of ROS levels was registered in Caco2 cells exposed to Fe-Si7_l-DOPA NPs. Furthermore, a similarity between the results obtained for a dose of 25 µg/mL Fe-Si 2_l-DOPA and Fe-Si2_CMC-Na samples, as well as of Fe-Si3_L_DOPA and Fe-Si3_CMC-Na ones, was observed, which suggests that ROS generation at a low dose of NPs is not influenced by the nature of stabilizer. However, it is clear that a 50 µg/mL dose of l-DOPA-coated Fe-Si NPs induced less ROS compared to CMC-Na-coated Fe-Si NPs. Moreover, we showed that stabilization of Fe-Si NPs with l-DOPA and CMC-Na reduced considerable the ROS production.

According to Zhou et al., studies [82], CMC-Na could play a major role as protective coating by decreasing the toxicity against microorganisms and oxidizing capacity of nanoscale zerovalent iron after suppressing the available oxidants from the surrounding media. 

In cancer cells, ROS levels are higher in comparison to normal cells, due to mitochondrial dysfunction, peroxisome activity, increased cellular receptor signaling, increased activity of oxidases, cyclooxygenases, lipoxygenases and thymidine phosphorylase [83]. The high rate of ROS production is counterbalanced by an equally high rate of antioxidant activity in cancer cells to maintain redox balance in order to ensure the cell survival. 

**Figure 10:**
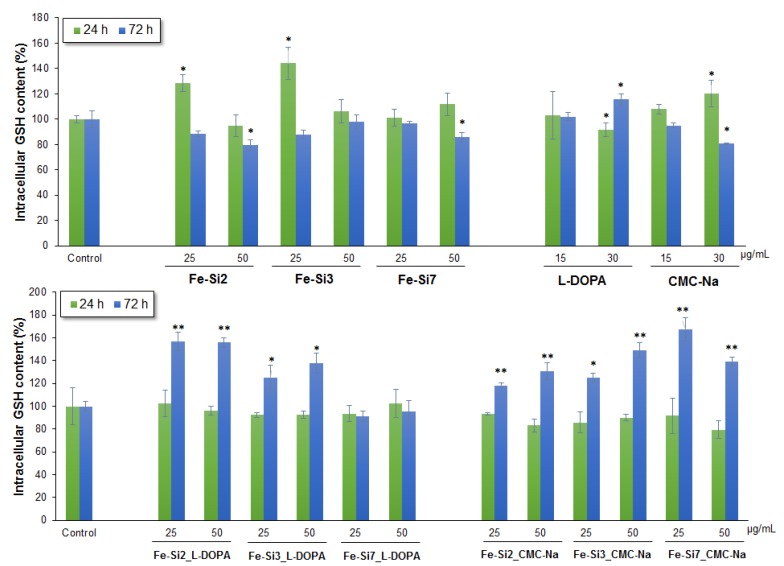
Intracellular GSH content in Caco2 cells after 24 and 72 h exposure to hybrid Fe-Si NPs stabilized with l-DOPA and CMC-Na. Data are expressed as the mean ± SD (*n* = 3) and represented as percentages of control. * *p* < 0.05; ** *p* < 0.01 versus control.

The concentration of reduced glutathione (GSH), a major component of cellular non-enzymatic antioxidant defense system, increased post-exposure in a time dependent manner. In the first 24 h, no significant variation of the GSH concentration was observed in Caco2 cells exposed to stabilized Fe-Si NPs, but a significant increase was detected in cells exposed to non-stabilized Fe-Si2 and Fe-Si3 NPs, and CMC-Na. After 72 h, its level rose significantly for all types of stabilized NPs, except Fe-Si7_l-DOPA (Figure 10). The highest level of GSH was found in Caco2 cells incubated with 25 µg/mL Fe-Si7_CMC-Na and Fe-Si2_l-DOPA, the percentage increases being 67% and 56% respectively. This suggests the activation of protecting mechanisms by elevating the stock supply of GSH, in order to face the increased ROS production, and thus, preventing the damage of lipids, proteins, and DNA caused by oxidative stress.

GSH is a major player in oxidative adaptation of cancer cells which is why it is exploited by researchers as a valid target for cancer targeted therapy [84]. The glutathione level is elevated in cancer cells in order to maintain the redox state and to protect cells from damage induced by free radicals, peroxides, and toxins. Glutathione is a powerful reducing compound, able to react with cellular toxic agents directly or via the reactions catalyzed by the glutathione *S*-transferase family of enzymes. Studies on NPs toxicity showed that GSH content could vary depending on NP dose. Increased GSH contents were reported by Saddick et al. [85] in brain tissue exposed to 500 μg/L Zn NP, while a significant decrease in GSH content was registered after exposure to 2000 μg/L. Similarly, Hao and Chen (2012) [86] found that GSH increased in the liver, gills, and brain of carp exposed to 0.5 and 5 mg/L of ZnO NPs, and decreased in all tissues of fish exposed to 50 mg/L ZnO NPs. Manke et al. [87] studied oxidative stress as an underlying mechanism for NP toxicity, and postulate that overexpression of antioxidant enzymes is indicative of mild oxidative stress, whereas mitochondrial apoptosis is induced during conditions of severe oxidative stress. According to the abovementioned, we can conclude that a mild oxidative stress was induced by 25 and 50 μg/mL stabilized Fe-Si NPs in Caco2 cells, which results in GSH level increase, and no cell death. During conditions of mild oxidative stress, transcriptional activation of phase II antioxidant enzymes occurs via nuclear factor (erythroid-derived 2)-like 2 (Nrf2) induction, another important mechanism by which cancer cells ensure their antioxidant protection. This transcription factor binds to antioxidant response element (ARE), and activates defensive gene expression, thus increasing the antioxidant proteins levels and protecting against oxidative damage [88]. Normally, Nrf2 interacts with Kelch-like ECH-associated protein 1 (KEAP1), thus leading it to proteasomal degradation. Elevated ROS levels oxidize redox sensitive cysteine residues on KEAP1, which cause the dissociation of KEAP1 from Nrf2, resulting in the increase of active Nrf2 level in the cytoplasm.

**Figure 11:**
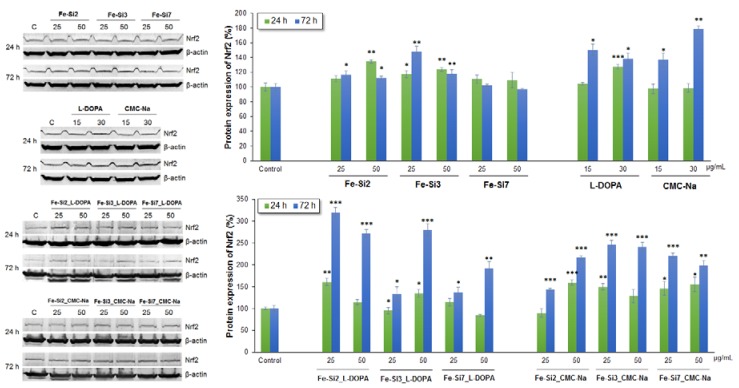
Relative protein expression of Nrf2 in Caco2 cells after 24 and 72 h exposure to 25 and 50 µg/mL of hybrid Fe-Si NPs stabilized with l-DOPA and CMC-Na. The images from the left represent the blot membranes for Nrf2 and β-actin at 24 and 72 h. The graph from the right represents the correspondent quantification of Nrf2 protein bands normalized to β-actin. Each set of samples was related to the untreated control migrated on the same gel. Data are expressed as the mean ± SD (*n* = 3) and represented as percentages. * *p* < 0.05; ** *p* < 0.01; *** *p* < 0.001 versus untreated control.

The Nrf2 protein expression was assessed in Caco2 cells after exposure for 24 and 72 h to 25 and 50 µg/mL of non-stabilized and stabilized hybrid Fe-Si NPs (Figure 11). In Caco2 cells, the activation of Nrf2 was time-dependent. After 24 h incubation, an increase of Nrf2 level in cells exposed to 25 µg/mL Fe-Si2_l-DOPA, Fe-Si3_l-DOPA, Fe-Si3_CMC-Na, Fe-Si7_CMC-Na samples, and to 50 µg/mL Fe-Si2_CMC-Na, Fe-Si3_l-DOPA, Fe-Si7_CMC-Na samples, compared with untreated control, was noticed. No change was registered for Fe-Si7 and Fe-Si7_l-DOPA samples. These results were in correlation with ROS and GSH levels. After 72 h, the increase of Nrf2 protein expression was significant in cells treated with both doses of all types of NPs. Furthermore, these results were in accordance with the increase of GSH content. By comparison, in cells exposed to the non-stabilized Fe-Si NPs and both stabilizers, the Nrf2 expression was slightly increased in accordance with the low GSH levels. 

In this study, we showed that the coating of Fe-Si NPs with l-DOPA or CMC-Na influenced the in vitro biological response in Caco2 cells. The effects of the individual components of the NPs were also investigated in tandem, and were different from those of stabilized Fe-Si NPs, most likely due to their low dispersion in cell culture medium and low internalization into Caco2 cells. According to the cell viability test results, the coating of Fe-Si NPs with l-DOPA increased the biocompatibility of NPs compared to individual components, whereas the capacity of Fe-Si NPs to induce cell death was not changed after coating with CMC-Na. However, instead, this combination reduced significantly the LDH leakage. The coating of hybrid Fe-Si NPs had a great influence on NP dispersion in the cell culture medium and on cellular internalization. The CMC-Na-coated NPs were far better dispersed compared with those stabilized with l-DOPA, which could explain their higher toxicity. Changes of F-actin cytoskeleton organization were highlighted also in the cells treated with CMC-Na-coated NPs, as well as with uncoated NPs and l-DOPA. After treatment with 25 and 50 µg/mL stabilized Fe-Si NPs, ROS were produced in Caco2 cells and a mild oxidative stress was most likely induced. Consequently, the GSH level increased in order to counteract the oxidative stress, which was in accordance with the increased Nrf2 protein expression. Biological data indicated also that Caco2 cells are able to deal with the effects induced by Fe-Si NPs at a dose below 50 µg/mL by activating the scavenging mechanisms against ROS production. Thus, we showed that the Caco2 biological response to Fe-Si NPs displays hormesis, which is an adaptive response of cells to a moderate stress. By comparison, in Caco2 cells treated with unstabilized Fe-Si NPs, a higher amount of ROS was generated, but a lower antioxidant response was induced. 

Studying of NP toxicity is a significant challenge. Numerous studies have appeared in the literature to differentiate between the toxicity of uncoated and coated NPs, which is extremely difficult, due to the diversity of factors influencing NP toxicity, such as size, shape, surface chemistry, synthesis techniques, coating agents, types of tissues/cells, etc. Similarly with our study, several papers found a higher toxicity of uncoated NPs than coated NPs [89], but some research studies have noticed the toxicity of uncoated NPs to be less than of the coated ones, which was associated with the NP cellular interaction and uptake [90]. Other researchers showed that the coating of iron NPs could decrease the leaching of iron ions and the lysosomal degradation of iron ions, thus reducing the oxidative stress and alterations in iron homeostasis [91,92]. 

A potential advantage of these new Fe-Si nanoparticle aggregates came from their dual composition: the iron-base phases gives them the sensitivity to magnetic fields which open the path for magnetic-guided/trapped drug delivery or variable-magnetic field-induced hyperthermia and/or heat-controlled drug release, whereas the luminescent nanosized silicon particles allow them to be simultaneously used for bio-labeling. Moreover, the silicon quantum dot toxicity is known to be much lower when compared with classical quantum dots, such as CdSe, CdTe, CdS, ZnS, or ZnSe [93], as proved for PEGylated micelle-encapsulated Si quantum dots synthesized also by laser pyrolysis, allowing them to be used for multiple in vivo applications, such as tumor vasculature targeting, sentinel lymph node, and multicolor near-infrared (NIR) imaging mapping [94]. The possibility of coupling Fe-based magnetic and Si fluorescent nanoparticles in a biocompatible probe was already proved in [95] by co-encapsulation of hydrophobized Fe_2_O_3_ superparamagnetic NPs and alkyl-capped Si quantum dots in PEGylated phospholipid micelles, yet in this case, those two functions can be uncoupled once the micelle is degraded. In our case, there are some aggregates in which the Fe-based and Si nanoparticles are strongly linked, and more difficult to break apart, which keeps connected, the magnetism and luminescence properties in biological media. However, the problem of luminescence loss in the aqueous-based biological fluids, due to the advanced oxidation of Si nanoparticles, still need to be countered by future studies for the founding of proper capping agents, and also by testing in this direction the CMC-Na and l-DOPA-coated NP aggregates reported in this work.

We think that the knowledge on toxicity of NPs is still limited, and much attention is required on development of new strategies and methods to explore the toxicity of all kind of NPs, in order to keep up with the rapid progress in the synthesis of novel nanomaterials for clinical applications.

## 4. Conclusions 

Magnetic nanopowders containing both Fe-based phases and silicon nanocrystals have been synthesized by one-step laser pyrolysis method, using separate, yet nearby flows of Fe(CO)_5_/C_2_H_4_ and SiH_4_/Ar, respectively, followed by their gentle oxygen-induced oxidative passivation. Their elemental composition was varied (in the sense of the silicon content decreasing) by keeping constant the Fe precursor flow and diminishing the silane-containing flow. The nanopowders were successfully ultrasonically dispersed in water-based media using two different biocompatible stabilizers: l-DOPA amino acid and Na carboxymethylcellulose polyelectrolyte.

Biological studies showed that l-DOPA-stabilized Fe-Si NPs tend to form aggregates in culture medium, thus minimizing their interaction with the cells, and also toxicity in comparison with those stabilized with CMC-Na that were more dispersed and toxic. However, the Caco2 cells were able to counteract the toxic effects by activating the cellular protection mechanisms. The coated hybrid Fe-Si NPs were found to be biocompatible with colon cancer cells at a dose below 50 µg/mL, and their biological responses were dependent on particle synthesis, surface coating, doses, and incubation time.

Therefore, the next generation of Fe-Si NPs with a biocompatible coating might be, in the future, functionalized with peptides targeting specific cancer cells for imaging studies, as well as local hyperthermia.

## Figures and Tables

**Table 1:** Experimental parameters for the standard/rapid quenching laser-assisted synthesis of Fe-Si-based hybrid nanoparticle aggregates.

Sample/Parameters	Exhaust Geometry	D1 Flow	D2 Flow		Laser Power	Flame Temp. (°C)
		D_C2H4_/Fe(CO)_5_ (sccm)	D_SiH4_ (sccm)	D_Ar_ (sccm)	P_L/Ar_/P_L/abs_. (W)	
Fe-Si2	normal	60	20	50	115/105	660
Fe-Si3	normal	60	10	40	115/108	620
Fe-Si7	narrow	60	5	55	115/110	540

**Table 2:** Elemental composition of powders extracted from EDX analyses.

Element/Sample	C (atom %)	O (atom %)	Si (atom %)	Fe (atom %)
Fe-Si2	11.1	23.8	34.3	30.8
Fe-Si3	13.4	33.1	26.7	26.8
Fe-Si7	13.3	26.1	19.9	40.7

**Table 3:** Magnetic parameters (saturation magnetization, retentivity, coercivity, and maximum permeability) of the raw powders.

Sample	M_s_ (emu/g)	M_r_ (emu/g)	H_c_ (kA/m)	µ_max_ (emu/Oe)
Fe-Si2	21	2.85	5.37	5.22 × 10−4
Fe-Si3	23	3.81	8.84	7.38 × 10−4
Fe-Si7	47	10.53	12.88	7.87 × 10−4

**Table 4:** IC_50_ values (doses that inhibits 50% of the cell viability) calculated after 72 h exposure of Caco2 cells to various doses of hybrid Fe-Si nanoparticles (NPs) and stabilizers ranging from 0–200 µg/mL and 0–120 µg/mL respectively. Data are expressed as mean concentration ± SD (*n* = 3).

NP Sample	IC_50_ (µg/mL)
Fe-Si2	382.18 ± 10.06
Fe-Si3	383.08 ± 11.86
Fe-Si7	904 ± 15.02
l-DOPA	81.2 ± 4.81
CMC-Na	1339 ± 14.27
Fe-Si2_l-DOPA	355.86 ± 17.23
Fe-Si3_l-DOPA	247.73 ± 14.56
Fe-Si7_l-DOPA	682.37 ± 14.07
Fe-Si2_CMC-Na	219.10 ± 11.83
Fe-Si3_CMC-Na	153.48 ± 8.14
Fe-Si7_CMC-Na	124.10 ± 7.10

**Table 5:** Intracellular ROS formation in the presence of hybrid Fe-Si NPs in Caco2 cells. Results are expressed as mean relative fluorescence units (RFU) ± SD of three independent experiments. * *p* < 0.05; ** *p* < 0.01 versus control.

Sample	Dose (µg/mL)	ROS Production (RFU)			
		1 h	2 h	3 h	4 h
Control	0	14.26 ± 0.98	18.46 ± 3.45	20.67 ± 1.23	22.15 ± 1.34
Fe-Si2	25	35.45 ± 3.2 **	63.19 ± 5.36 **	78.32 ± 6.49 **	82.37 ± 6.96 **
	50	37.63 ± 2.06 **	67.93 ± 4.92 **	84.08 ± 5.5 **	88.79 ± 5.7 **
Fe-Si3	25	39.59 ± 3.42 **	70.43 ± 6.14 **	88.83 ± 6.38 **	92.41 ± 7.63 **
	50	45.87 ± 10.6 *	81.56 ± 17.35 *	101.3 ± 20.49 *	106.46 ± 22.26 *
Fe-Si7	25	47.58 ± 5.76 **	80.44 ± 9.76 **	93.68 ± 11.33 **	101.24 ± 12.00 **
	50	56.02 ± 8.8 *	91.74 ± 12.63 *	104.05 ± 12.82 **	114.15 ± 14.82 **
L-DOPA	15	11.83 ± 0.48	16.93 ± 0.78	18.73 ± 1.13	20.32 ± 1.04
	30	11.65 ± 0.62	16.63 ± 1.62	18.3 ± 1.99	19.91 ± 2.02
CMC-Na	15	13.04 ± 0.59	19.36 ± 1.06	23.02 ± 1.74	24.43 ± 1.78
	30	11.28 ± 0.62	16.85 ± 0.77	19.73 ± 0.86	21.1 ± 1.00
Fe-Si2_L-DOPA	25	18.39 ± 1.85 *	25.76 ± 2.35 *	29.84 ± 2.99 *	33.03 ± 3.26 *
	50	13.28 ± 1.08	18.23 ± 1.73	21.15 ± 1.89	23.79 ± 1.98
Fe-Si3_L-DOPA	25	19.22 ± 0.42	27.64 ± 0.99 *	32.91 ± 1.67 **	36.36 ± 2.20 **
	50	14.81 ± 2.62	20.19 ± 3.92	22.86 ± 4.26	25.28 ± 4.85
Fe-Si7_L-DOPA	25	19.23 ± 2.47	26.52 ± 3.26	31.17 ± 4.02	35.02 ± 4.70
	50	15.11 ± 1.24	21.23 ± 1.94	24.42 ± 2.23	27.07 ± 2.56
Fe-Si2_CMC-Na	25	16.56 ± 1.50	23.78 ± 2.11*	28.69 ± 2.39*	32.34 ± 2.45 **
	50	15.53 ± 1.69	22.71 ± 2.57	28.36 ± 3.24*	32.68 ± 3.74 *
Fe-Si3_CMC-Na	25	17.51 ± 1.02 *	25.5 ± 1.48 *	31.4 ± 1.82 **	35.9 ± 2.04 **
	50	19.71 ± 0.60 *	29.52 ± 0.95 *	37.7 ± 1.27 **	43.68 ± 1.40 **
Fe-Si7_CMC-Na	25	23.59 ± 3.00 *	39.43 ± 4.53 **	50.29 ± 5.77 **	58 ± 6.61 **
	50	25.23 ± 1.75 *	42.66 ± 3.44 **	56.44 ± 4.65 **	67.36 ± 6.22 **
